# Sensory feedback modulates Weber's law of both perception and action

**DOI:** 10.1167/jov.24.13.10

**Published:** 2024-12-17

**Authors:** Ailin Deng, Evan Cesanek, Fulvio Domini

**Affiliations:** 1 Department of Cognitive Linguistic & Psychological Sciences, Brown University, Providence, RI, USA; 2 Mortimer B. Zuckerman Mind Brain Behavior Institute, Columbia University, New York, NY, USA

**Keywords:** weber's law, sensorimotor adaptation, grip aperture, manual estimation, action

## Abstract

Weber's law states that estimation noise is proportional to stimulus intensity. Although this holds in perception, it appears absent in visually guided actions where response variability does not scale with object size. This discrepancy is often attributed to dissociated visual processing for perception and action. Here, we explore an alternative explanation: It is the influence of sensory feedback on motor output that causes this apparent violation. Our research investigated response variability across repeated grasps relative to object size and found that the variability pattern is contingent on sensory feedback. Pantomime grasps with neither online visual feedback nor final haptic feedback showed variability that scaled with object size, as expected by Weber's law. However, this scaling diminished when sensory feedback was available, either directly present in the movement (Experiment 1) or in adjacent movements in the same block (Experiment 2). Moreover, a simple visual cue indicating performance error similarly reduced the scaling of variability with object size in manual size estimates, the perceptual counterpart of grasping responses (Experiment 3). These results support the hypothesis that sensory feedback modulates motor responses and their associated variability across both action and perception tasks. Post hoc analyses indicated that the reduced scaling of response variability with object size could be due to changes in motor mapping, the process mapping visual size estimates to motor outputs. Consequently, the absence of Weber's law in action responses might not indicate distinct visual processing but rather adaptive changes in motor strategies based on sensory feedback.

## Introduction

### Weber's law and its apparent violation in grasping

Weber's law is a psychophysical principle widely observed across sensory modalities and perceptual dimensions (Fechner, 1965; Weber, 1831). It states that discrimination thresholds are proportional to the intensity of physical stimulus. This law is typically studied through the measurement of the just noticeable difference (JND), the most commonly used discrimination threshold and defined as the smallest change in stimulus magnitude that yields a perceptible change in estimate, usually assessed via comparison tasks.

#### Seminal study on Weber's law in grasping

The study of Weber's law was traditionally performed with perceptual tasks where deriving estimations is straightforward. When Ganel and colleagues extended the study of Weber's law to the realm of action as an attempt to investigate the estimation for visually guided action, revisions were required. They measured the maximum grip aperture (MGA)—the largest separation between the grasping fingers along the movement trajectory—as the response reflecting the size estimate of the target object used for motor planning (Ganel, Tanzer, & Goodale, 2008; Jeannerod, 1986; Paulignan, Frak, Toni, & Jeannerod, 1997). Critically, they used the within-subject standard deviation of MGAs toward the same object as (a proxy for) the JND for that object. Observing that this MGA variability remained unchanged with object size, they concluded that the visual estimation for action eludes Weber's law (Ganel, Chajut, & Algom, 2008).

As control conditions, three additional tasks were conducted: a manual size estimate (MSE) task, where subjects adjusted the distance between their thumb and index finger to match the perceived size of an object while keeping their hand position stationary; an adjustment task, where subjects adjusted a stimulus on a computer screen to represent the perceived object size; and a memory-based delayed grasping, where the reach-to-grasp was delayed for 5 seconds after withdrawing the vision of the object. Critically, in all three control tasks, the response variability scaled with object size, in accordance with Weber's law. These results indicated that the response variability associated with (real-time) visually guided actions is indeed uncharacteristic.

#### Two-visual stream explanation

Ganel and colleagues explained the absence of Weber's law in grasps based on the influential two visual streams hypothesis (TVSH), which proposes separated visual streams or processing pathways (Goodale & Milner, 1992; Westwood & Goodale, 2011). These pathways compute shape information differently. According to this explanation, the visual processing for visually guided grasps primarily relies on the dorsal stream, which encodes information using absolute metrics and thus produces shape estimates that violate Weber's law. In contrast, the visual processing for other tasks primarily relies on the ventral stream, which encodes information using relative metrics and thus produces shape estimates that adhere to Weber's law. Therefore, adherence to Weber's law depends on whether the ventral visual processing is employed for the task.

This seminal study and its explanation have been widely accepted, with its methods being applied and generalized in subsequent studies. It has become common for researchers to use the pattern of response variability to infer which visual processing stream^1^ is employed. For example, Heath and colleagues (Heath, Holmes, Mulla, & Binsted, 2012) examined the variability of the grip aperture at different time points throughout the grasping trajectory. With Weber's law being present in the early stage but absent later, they suggested that a grasp is initially driven by the ventral stream, but the dorsal stream seizes control as the action unfolds. Similarly, because Weber's law has been detected in “non-conventional” grasps, such as two-dimensional (2D) object grasps (Holmes & Heath, 2013; Hosang, Chan, Davarpanah Jazi, & Heath, 2016; Ozana & Ganel, 2019) or pantomime grasps^2^ (Holmes, Marriott, Mackenzie, Sin, & Heath, 2012; Davarpanah Jazi, Yau, Westwood, & Heath, 2015), it has been concluded that these grasps are primarily guided by the ventral stream.

#### Alternative explanations based on mechanisms that do not involve visual processing

Notably, distinctions between perceptual and action tasks may arise from factors beyond visual computation. Consider the differences between manual estimation and grasping. Although the tasks share the end effector (i.e., both responses manifest as finger apertures), their responding processes are distinct. Manual estimation, highly dependent on proprioception, aims to match the perceived object size. In contrast, grasping incorporates a broader range of sensory feedback and more versatile feedback mechanisms; furthermore, as it involves physical interaction with the object, the goal shifts from simply matching object size to optimizing movement paths and achieving desired motor outcomes. This requires MGA to surpass object size, ensuring room for the fingers to close and secure a firm grip (Smeets, van der Kooij, & Brenner, 2019). Consequently, MGA is typically larger than MSE.

An alternative explanation for the dissociation in Weber's law has been proposed based on these task specific differences (e.g., requirements, constraints). This explanation centers on the biomechanical limit of digit span (Utz, Hesse, Aschenneller, & Schenk, 2015): As finger apertures approach this biomechanical limit, further increase becomes progressively more difficult. This ceiling effect, which reduces variability as apertures increase, counteracts the expected pattern of Weber's law. Given that MGA often reaches closer to this biomechanical limit due to its larger size, it is more affected by this ceiling effect than MSE. Consequently, MGA and MSE exhibit dissociated patterns of variability.

This explanation provides a logical and testable mechanism; however, it is still under debate whether this explanation alone can fully account for the observed discrepancies in variability pattern across tasks (Ayala, Binsted, & Heath, 2018; Bruno, Uccelli, Viviani, & De'Sperati, 2016; Heath & Manzone, 2017). Moreover, compared with the explanation based on dissociated visual processing, this explanation is less generalizable, as it struggles to account for other characteristics unique to action responses, such as their robustness against visual illusions (Aglioti, DeSouza, & Goodale, 1995; Ganel et al., 2008; Haffenden, Schiff, & Goodale, 2001).

### Reassessing JND and Weber's law in motor response

The just noticeable difference refers to the smallest detectable difference between two sensory stimuli that a person can perceive. Traditionally, JND is measured through discrimination tasks such as the two-alternative forced choice paradigm, where two stimuli are presented either sequentially or side by side, and the participant must choose which one has the greater (or lesser) magnitude of the property being tested. More broadly, JND serves as a measure of estimation resolution, reflecting the precision of the sensory system in distinguishing differences. In some contexts, JND is derived from tasks that involve reproducing internal representations, such as the icon probe adjustment method; however, when testing grasping, JND is approximated using the standard deviation of motor outputs. This approach is somewhat controversial and remains under debate: Motor outputs may not precisely reflect visual estimates (Bhatia, Löwenkamp, & Franz, 2022; Schenk, Utz, & Hesse, 2017; Smeets & Brenner, 2008). As discussed in the previous text, rather than matching object size, MGA in grasp is more about preparing for a firm final grip or other desired outcomes. Additionally, considerable motor noise contributes to the variability in motor outputs, making it a less reliable indicator of estimation resolution.

Given the unresolved ambiguities, we adopt a provisional definition in this paper: Weber's law is present in a task *if the standard deviation of the responses scales with the stimulus magnitude*. For example, in the context of grasping movements, if the variability of MGA increases with object size, then Weber's law is considered to be present.

It is important to note that this definition is conditional, tailored to facilitate the current discussion on the variability of motor response. It captures only an apparent characteristic of motor response, and interpretations should be deferred.

### Role of sensory feedback

Among the distinctions across the tasks, sensory feedback stands out as a universal factor that differentiates actions from other tasks. Action typically incorporates more sensory feedback. For example, when you reach to grasp your cell phone on a desk, you can rely on vision to avoid bumping into other objects or missing the target while reaching forward or to adjust your fingers for a secure final hold as your hand nears the phone. Upon physical contact, you receive tactile feedback. Thus, compared with simply viewing, you get online visual feedback and final haptic feedback when you grasp it. The feedback greatly changes the movement dynamics, affecting the movement outcomes. Therefore, the availability of feedback in behavioral tasks has been the focus of a number of investigations.

#### Reported influence of visual feedback

By online visual feedback, we refer to the real-time visual information of the hand relative to the object being grasped, which provides immediate information about their relative distance and velocity. By this definition, in the pantomime grasping that is directed to the area adjacent to the target object, vision during movement does not support valid online visual feedback, as the relative distance or velocity between the hand and the virtual target is not immediately available (Jazi & Heath, 2017).

Studies on Weber's law in motor responses typically have focused on the visual estimate used to program the movement. To avoid interference from real-time adjustments, vision has usually not been permitted during the movement phase. As a result, the effect of online visual feedback on adherence to Weber's law has rarely been investigated. One notable exception is a study by Heath, Mulla, Holmes, and Smuskowitz (2011), which compared closed-loop grasping (with online vision) to open-loop grasping (without online vision) to examine Weber's law. As Weber's law was absent in both conditions, the authors concluded that online visual feedback did not significantly impact adherence to Weber's law. However, haptic feedback was present in both conditions, likely influencing adherence and masking the effects of visual feedback (see text below). Consequently, the isolated effect of visual feedback remains unexplored.

#### Reported influence of haptic feedback

Final tactile contact with the physical object provides haptic information about object size, distinguishing grasping from other tasks. To equate the availability of haptic feedback for grasping and manual size estimation, in the original study by Ganel et al. (2008) and subsequent studies (Heath et al., 2011; Holmes, Mulla, Binsted, & Heath, 2011; Löwenkamp, Gärtner, Haus, & Franz, 2015), subjects were asked to reach to grasp the target object after completing each manual estimation trial. Yet, Weber's law was still apparent in the manual estimation task.

In contrast, other studies have reported that haptic feedback significantly influences non-conventional grasping (Davarpanah Jazi et al., 2015; Hosang et al., 2016; Jazi & Heath, 2017). In those studies, when grasps were performed without physical interaction with three-dimensional (3D) objects, they adhered to Weber's law. But, when additional haptic feedback was provided (by placing a physical object between the fingers after completing the grasp), the grasping response violated Weber's law. Interestingly, the study by Jazi and Heath (2017) further suggested that the mere presence of haptic feedback did not guarantee this effect: Although the additional haptic feedback eliminated Weber's law in the pantomime grasping targeted at the object (with the object removed from that location before the onset of movement), it failed to affect Weber's law adherence in pantomime grasping directed at an area adjacent to the object.

#### Objective of this study

To our knowledge, the effect of haptic feedback in eliminating Weber's law has only been explained through dissociated visual processing, proposing a switch from ventral stream processing (associated with general perception using relative encoding) to dorsal stream processing (specified for real-time action using absolute encoding). However, the explanation has not been directly tested, and the underlying mechanisms behind this transition remain unknown. The current study aimed to investigate these mechanisms.

**Figure 1.**
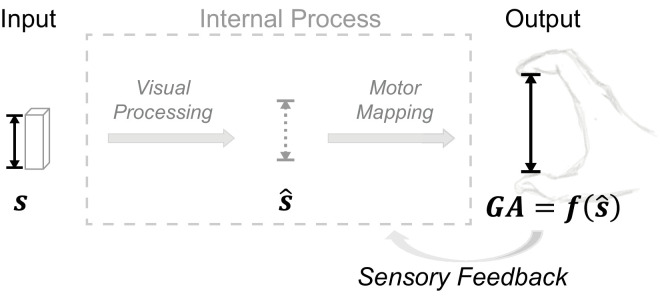
We proposed two hypotheses on how sensory feedback influences motor output regarding its adherence to Weber's law: a vision-based hypothesis and a motor-based hypothesis. To contrast these hypotheses, we modeled the process of generating task responses as a cascade of two stages. The first stage is visual processing, which computes the size estimate *ŝ* from the visual input of an object of size *s*. The second stage is motor mapping, translating the visual estimate into the motor output *GA* through a function *f*(*ŝ*). The vision-based hypothesis emphasizes changes in the first stage of visual processing, whereas the motor-based hypothesis highlights the influence of sensory feedback on the second stage of motor mapping.

Specifically, we formulated two competing hypotheses through which sensory feedback affects the adherence of motor outputs to Weber's law (see Figure 1).

##### Vision-based hypothesis

This hypothesis assumes two modes of visual processing: the perception mode, which yields visual estimates adhering to Weber's law, and the action mode, which yields estimates that escape Weber's law. Because sensory feedback is closely related to action, its presence promotes the engagement of the action mode.

##### Motor-based hypothesis

Sensory feedback in action significantly alters motor control dynamics by enhancing state estimations (e.g., position, velocity). This improves error detection, prompting both fast online corrections and slower trial-by-trial adjustments, such as sensorimotor adaptation. Over multiple movements, these feedback-induced adjustments/adaptations can lead to notable changes in movement planning and execution, driving action responses to be increasingly distinct from other task responses. This hypothesis proposes that the absence of Weber's law in grasping responses is a result of the specific motor mapping shaped under the influence of sensory feedback.

##### Vision-based versus motor-based hypotheses

Vision-based versus motor-based hypotheses differ in several key aspects:

• The vision-based hypothesis requires the assumption of task-dependent visual processing, with visual processing for grasping (or action in general) being distinct from that of other tasks. In contrast, the motor-based hypothesis postulates the same visual computations across tasks.
• The motor-based hypothesis relies on sensory error signals to drive changes in motor mapping. In other words, this hypothesis requires sensory feedback to provide effective error signals to initiate any change, whereas the vision-based hypothesis does not.
• The vision-based hypothesis expects changes triggered by sensory feedback to occur instantaneously, with the visual estimate and resulting motor response immediately shifting from Weber's law adherence when feedback is absent to Weber's law violation when feedback is present. In contrast, the motor-based hypothesis expects changes to take longer, occurring over multiple movements.

### Overview of the current study

The current study aimed to investigate how sensory feedback influences motor responses regarding its adherence to Weber's law. To complement previous studies based on the vision-based hypothesis, we examined a novel motor-based hypothesis. This hypothesis posits that sensory feedback affects the late stage of motor actions rather than modulating visual processing (see Figure 1). Specifically, we hypothesized that sensory feedback enhances error detection and motor adaptation, altering motor mapping in such a way that the resulting response variability no longer scales with object size, or at least does so to a lesser extent. In order to facilitate the detection of sensory error signals for both grasping and manual estimation, we adopted experimental conditions that differed from most previous studies in the following ways:

• The visual stimulus remained visible throughout the trial, including during the grasping movement or during finger adjustments in manual estimation. This contrasts with most previous experiments, which did not allow vision after the preview of the stimulus (see previous text for details).
• All tasks were performed at/toward the same location where the visual stimulus was presented. This setup, typically applied to grasping physical objects only (e.g., previous manual estimations were performed at a different location from the stimulus), was applied consistently across tasks.
• Manual size estimations were performed directly upon the visual stimulus, with feedback provided immediately after each estimate. This contrasts with previous experiments where the estimation was performed at a different location than the stimulus, and haptic feedback was delivered only after an additional grasping movement.

#### Experiments overview

1. Experiment 1 examined Weber's fraction of grasps as we varied the availability of online visual feedback and final haptic feedback. We predicted that the condition in which no feedback was provided (pantomime grasps) would adhere to Weber's law, whereas the presence of sensory feedback (visual, haptic, or both) would reduce Weber's fraction or eliminate Weber's law.
2. Experiment 2 examined whether sensory feedback could reduce Weber's fraction in pantomime grasps. We compared pantomime grasps executed within the same block to those intermixed with grasps where both visual and haptic feedback were available.
3. Experiment 3 explored whether sensory feedback could reduce Weber's fraction in manual estimation by delivering visual feedback immediately after completing each estimate.

## Experimental setup and stimuli

### Apparatus

**Figure 2.**
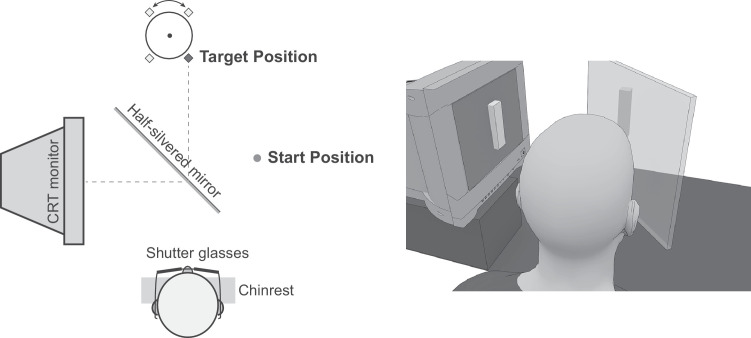
Experimental setup. (Left) Top–down view of the visual display system and the haptic device. (Right) A rendered scene illustrating the presentation of the visual target object.

Experiments were executed in an augmented-reality system that allows the independent manipulation of the visual and the haptic presentation of a target object. A computer-generated visual stimulus was presented through mirror reflection, and the haptic stimulus was precisely overlaid onto the visual stimulus (see Figure 2).

The apparatus consisted of a 19-inch cathode-ray tube (CRT) monitor, a pair of NVIDIA 3D Vision 2 wireless glasses (NVIDIA Corporation, Santa Clara, CA), a half-silvered mirror arranged at a 45° angle to the monitor, and a motorized carousel holding physical objects. The monitor positioned to the left of the participant displayed the designated stereoscopic 3D object that, through reflection on the 45° mirror, simulated a 3D object located in front of the participant. The room remained dark during experimental blocks to exclude visual interference. In order to ensure that the stimulus was viewed from the correct viewpoint, the participant's head movement and position were restricted with a chin rest. To provide haptic feedback, the matching physical object was rotated to the exact location where the 3D virtual object was simulated.

Finger movement was tracked at 85 Hz using the Optotrak Certus motion-capture system (NDI, Waterloo, CA). Lightweight posts with Optotrak infrared markers were attached to thumb and index fingernails with sticky tack. With three tracking markers on each finger, we tracked not only the position but also the orientation of the finger segment between the fingertip and the first joint.

### Stimuli

The stimuli set consisted of four cuboids with the same width and depth (12.8 mm) and varying heights: 21 mm, 28 mm, 35 mm, and 42 mm. One stimulus from the set was tested at a given trial, which was presented at a fixed target location 380 mm straight ahead. The visual stimulus appeared as outlines of a computer-generated vertical cuboid rendered stereoscopically. The physical stimulus, providing haptic feedback of the height, was a 3D-printed block matching the visual stimulus in height and depth but with a width of 19.2 mm. This width increase, an adjustment based on pilot study observations, ensured a firm grip even when the orientation of the fingers was slightly misaligned with the (vertical) grasping axis and rotated around the line of sight. These physical blocks were attached to a carousel, controlled by the experimental program, which could move and/or rotate to position the objects at the designated target location (Figure 2, left).

### Sensory feedback

#### Online visual feedback

**Figure 3.**
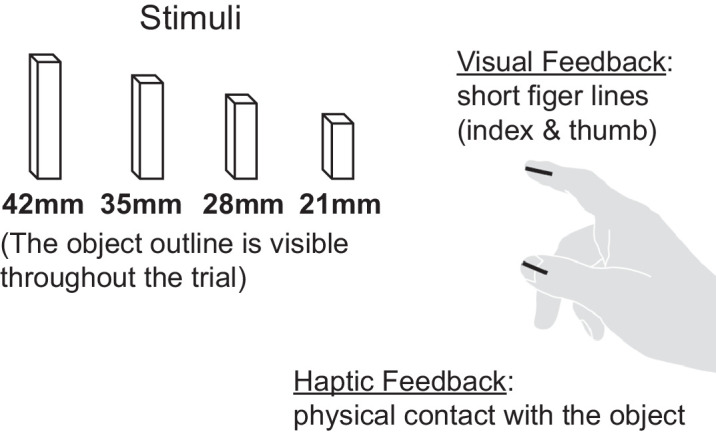
The target object was chosen from a set of four cuboids with the following dimensions along the grasping axis: 21 mm, 28 mm, 35 mm, or 42 mm. We independently presented two types of sensory feedback. When visual feedback was available, the participant saw 3D line segments representing the real-time position and orientation of their thumb and index. When haptic feedback was available, the participant had tactile contact with a physical object that overlaid the visual target.

The online visual feedback was the real-time presentation of sections of thumb and index: 3D lines representing the segments between the fingertip and the first joint (see Figure 3). Therefore, when the visual feedback was available, participants experienced in real time the visual segments as belonging to their actual fingers.

**Figure 4.**
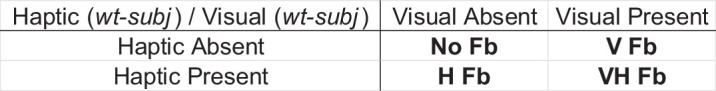
This table shows the testing conditions of the experiment. Each subject performed four blocks of grasps, each corresponding to one cell from the table. The block order was counterbalanced for all subjects.

#### Final haptic feedback

Haptic feedback refers to the tactile contact with the physical object at the end of the grasping movement. When haptic feedback was available, the carousel transported the physical object to the target position before each trial. The participant would touch and hold the physical object for 1.5 seconds at the end of the movement. When haptic feedback was withdrawn, the carousel was pushed back and stayed out of the participant's reach, but it still rotated before each trial to control for the rotation time and sound.

### Tasks and responses

#### Reaching: training/distractor

Participants underwent training in reaching trials before engaging in the experimental trials across all experiments. During this training phase, they familiarized themselves with the basics of our setup, including the designated position of *home*. They learned to perform movements with finger-tracking devices and to return home after each movement. Additionally, in some experiments (Experiment 1 and the BLOCKED group in Experiment 2), these trials served as distractor trials between blocks, forcing participants to deviate from the movement patterns established in the previous block. This reduced the influence of sensory feedback from the previous block (Bozzacchi, Volcic, & Domini, 2014).

To perform the reaching task, participants started from home with their thumb and index finger closed together. Without visual feedback of their hand, they reached to touch a target cuboid while maintaining the pinched position of the thumb and index finger. Unlike the target objects used in other tasks, the target cuboid for reaching had randomized dimensions and was positioned at a random location within the participant's reachable space.

The reach would be deemed a “hit” if the fingertips were less than 20 mm from the cuboid center or a “miss” otherwise. The training was completed when the participant achieved five consecutive hits.

#### Precision grasp: Experiments 1 and 2

Starting from the designated home position and with the thumb and index closed together, the participant reached to grasp the target object along its vertical axis with their index finger and thumb. At the end of the movement, they held their fingers on the top and bottom surfaces of the object for 1.5 seconds until a beep signified trial completion. Trials would not proceed until the hand had returned home. As described above, the participant was asked to hold the top and bottom surfaces for 1.5 seconds at the end of the grip. It was straightforward when a physical object was available for haptic feedback. But, when haptic feedback was withdrawn, they were asked to keep the fingers in mid-air where they thought the correct grasp would take place. If the final grip was more than 20 mm from the target object, the grasp would be deemed a miss, and the participant would make a new attempt.

The MGA in each movement was taken as the response. MGAs from reattempted trials took less than 2% of all responses.

#### Manual size estimation: Experiment 3

Starting with the thumb and index closed together, participants opened and adjusted their right hand's grip until they felt it matched the height of the visual stimulus. No vision of the hand or grip was provided during the adjustment. A key press with the left hand entered their response. The grip aperture at the key press was taken as the MSE and the trial response.

### Key variables and analyses

#### Key variables and measures

The current study investigated the impact of sensory feedback on motor output and the apparent Weber's law. We used two primary variables for this investigation:

• Landmark grip aperture (GA*)
  – Grasping movements—MGA, the maximum finger separation throughout the grasping movement
  – Manual size estimation—MSE, the finger separation when confirming trial response
• Within-subject standard deviation of landmark grip apertures (SD_GA__*_)
  – Grasping movements—*SD_MGA_*, reflecting the variability of grasping response
  – Manual size estimation—*SD_MSE_*, reflecting the variability of the estimation task response

The measures of these two variables as a function of object size are particularly important:

• Slope of GA*—*k_MGA_* or *k_MSE_*, a key measure of the mapping function for the motor output, reflecting the motor output sensitivity to change of stimulus size
• Slope of SD_GA__*_—*W_MGA_* or *W_MSE_*, representing the apparent Weber's fraction based on our provisional definition of Weber's law, which is considered present if the variability of the motor response scales with object size (for details, see the earlier Reassessing JND and Weber's law in motor response section).

#### Initial processing

The landmark grip apertures were first processed within each subject. For subject *i*, we calculated the mean and standard deviation across all trials (repetitions) for feedback condition *FbCond* at object size *Size*, yielding $GA_{\left(i\right)}^{*}$ (*FbCond*, *Size*) and SD_GA__*__(_*_i_*_)_ (*FbCond*, *Size*), respectively. Furthermore, the measures within the same feedback condition were linearly fitted with object size to determine their slope measures, which yielded k_GA__*__(_*_i_*_)_ (*FbCond*) and W_GA__*__(_*_i_*_)_ (*FbCond*). At this point, we obtained the key measure estimates from each subject. These values served as the basis for further analyses and figures of aggregated results.

#### Estimated effect of feedback

We compared key variables across different conditions to assess how they varied in the presence of feedback with respect to the individual baseline with no feedback. When comparing two conditions, we relied on traditional paired *t*-tests to compare key variables and provide Cohen's *d* as a measure of effect size. For comparisons involving more than two levels, we employed linear mixed models that incorporated by-subject random intercepts and analysis of variance (ANOVA) to handle multilevel comparisons in a way analogous to the paired *t*-tests. Specifically, we fitted the data using the lme4 package in R (R Foundation for Statistical Computing, Vienna, Austria) (Bates, Mächler, Bolker, & Walker, 2015). The fitted coefficients reflect the direction and magnitude of changes as the feedback condition varied. We tested the main effect of the feedback condition using type III ANOVA (Wald χ^2^ test) from the R car package (Fox & Weisberg, 2019) and estimated the corresponding effect size using the R sjstats package (Lüdecke, 2024).

#### Determining the adherence of Weber's law

To determine whether Weber's law was present or absent, we performed Bayes factor *t*-tests on subjects’ Weber's fractions, W_GA__*_, using the point null hypothesis. We chose to use this type of test because it tests for both the presence and the absence of an effect. The Bayes factors reported earlier were based on the default alternative prior, a Cauchy distribution centered on zero with a scale of $\sqrt{2}/2$ (Morey & Rouder, 2023). Sensitivity analyses performed using rscales of 0.5 and 1.0 are included in the Appendices, along with results of traditional *t*-tests and Cohen's *d* effect sizes.

### Participants

#### Recruiting and screening

Participants were right-handed individuals with normal or corrected-to-normal vision recruited within the Brown University community. They received cash compensation ($12/hr) or class credits for participation. Informed consent was obtained from all participants prior to the experiment, in accordance with the protocol approved by the Brown University Institutional Review Board and performed in accordance with the tenets of the Declaration of Helsinki. After providing consent, the participants underwent a screening pretest with random-dot stereograms to evaluate their stereo acuity. Participants lacking stereo vision did not participate in the experiment.

#### Excluding outliers

Trials were excluded if the landmark grip aperture deviated by 2.5 *SD* or more from the subject's mean grip aperture, serving as a criterion not only to filter out anomalous movements but also to eliminate data compromised by finger tracking difficulties. These issues typically arose from infrared markers being occluded by other fingers or misaligned with the Optotrak. It was observed that movements from certain participants, under specific testing conditions, were more prone to these tracking difficulties. To address this, participants were identified as outliers and excluded from the analysis if any of their GA* or SD_GA__*_ values fell outside 2.5 *SD* from the mean value across subjects. This criteria were consistently applied to all experiments.

## Experiment 1: Effect of online visual feedback and haptic feedback on Weber's law in grasps

### Motivation

Although previous studies have shown that haptic feedback is effective in eliminating Weber's law in grasps, the effect of online visual feedback has not been systematically explored. The current experiment investigated the effect of sensory feedback on Weber's fraction of grasps as we independently manipulated the availability of online visual feedback and final haptic feedback.

### Design

We employed a 2 × 2 factorial within-subject design, incorporating two factors: presence or absence of online visual feedback and the presence or absence of final haptic feedback (as shown in Figure 4). Each subject completed four blocks of grasps, representing all possible combinations of the factors. Block order was randomized with a Latin square design.

### Methods

Each block started with reaching trials—the ones before the first block served as training, and the subsequent ones served as distractors to reduce carry-over effects. Participants took a 1- to 5-minute break between blocks.

Each block was comprised of 80 grasping trials, with 20 repetitions for each object size. The presentation order of the object sizes was pseudorandomized.

### Participants

Twenty five participants completed the experiment. Data from six outliers participants were eliminated based on the criteria detailed earlier. Notably, the outlier removal perturbed the Latin square randomization or the perfect balance of order, but the feedback conditions remained sufficiently randomized. For the data from the remaining participants, less than 4% of the responses were identified as outliers and removed.

### Results

**Figure 5.**
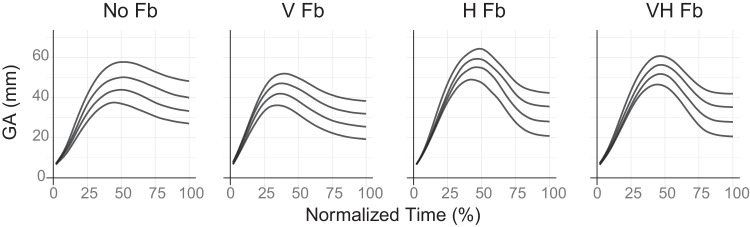
Average trajectories in Experiment 1 along normalized movement time. For each condition, the trajectories ordered from lowest to highest correspond to object sizes of 21 mm, 28 mm, 35 mm, and 42 mm.

**Figure 6.**
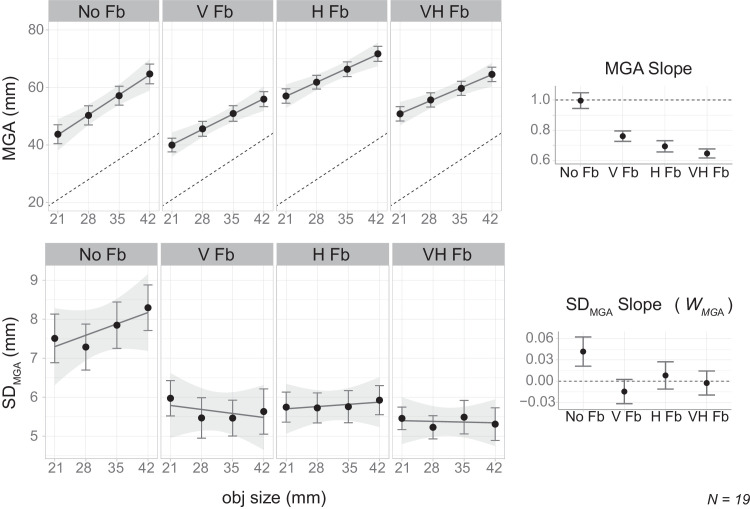
Aggregated results for Experiment 1. The top row shows results regarding MGA, and the bottom row shows results for *SD_MGA_*. For each row, the left panels present the key variable as a function of object size, separated by feedback conditions: no feedback (“No Fb”), visual feedback only (“V Fb”), haptic feedback only (“H Fb”), and both visual and haptic feedback (“VH Fb”); the rightmost panel presents the slope of that variable with respect to object size. Error bars indicate the standard errors of the mean across subjects.

Figure 5 illustrates variations in trajectories across different feedback conditions, suggesting that the availability and type of sensory feedback may influence motor output mapping functions. The upper left of Figure 6 points to a potential decrease in the magnitude of the response (MGA) when visual feedback was provided, whereas haptic feedback appears to increase it. However, both types of feedback reduced the slope of the response with respect to object size (see upper right of Figure 6 and Table C2 in Appendix C) and the magnitude of response variability (see lower left of Figure 6 and Table C3 in Appendix C).

**Figure 7.**
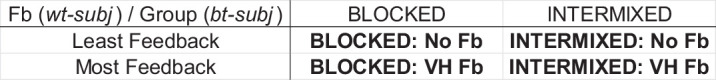
The table visualizes the mixed factorial design of Experiment 2. The within-subject factor was the presence or absence of sensory feedback. The between-subject factor was the blocking condition of trials: grasps with different feedback conditions were either blocked or intermixed.

#### Reduction of Weber's fraction in the presence of feedback

We calculated Weber's fraction for each subject in each condition and assessed how the fraction varied by feedback condition using linear mixed models with random intercepts. Table 1 presents the estimated values, including the baseline fraction for the no feedback (“No Fb”) condition and the estimated change in other conditions relative to this baseline. Although the omnibus test (comparing all four condition levels) did not yield a statistically significant main effect of condition (χ^2^(3) = 5.434, *p* = 0.143), the moderate effect size (η^2^ = 0.09) suggests that meaningful differences may exist across conditions.

**Table 1.** The Weber's fraction change was estimated by linear mixed model formulated as *WB_MGA_* *Cond* + (1 *subj*). It was fitted by restricted maximum likelihood (REML), and the *t*-tests used Satterthwaite's method.

	Estimated	*SE*	*df*	*t*	*p*
“No Fb” (intercept)	0.042	0.019	71.37	2.252	0.027*
“V Fb” − “No Fb”	−0.056	0.025	54.00	−2.213	0.031*
“H Fb” − “No Fb”	−0.034	0.025	54.00	−1.320	0.192
“VH Fb” − “No Fb”	−0.044	0.025	54.00	−1.741	0.087

Given that our hypothesis centers on the availability of feedback (whether feedback is present or absent, rather than its type), we further compared Weber's fractions between conditions with and without feedback using planned contrast for the prediction condition. The results indicated that the presence of feedback led to a statistically significant reduction in Weber's fraction, (χ^2^(1) = 4.737; *p* = 0.030; η^2^ = 0.08), with a mean decrease of 0.045 (*SE* = 0*.*021). Furthermore, Weber's fractions did not significantly differ among the three feedback conditions (*χ*^2^(2) = 0*.*892; *p* = 0*.*640; η^2^ = 0*.*02).

#### Weber's law adherence in each condition

As Figure 6 (lower right) illustrates, the slope of the *SD_MGA_* only appears to be above zero in the condition with no sensory feedback, suggesting the adherence to Weber's law in that specific case.

In the conditions with sensory feedback, test results suggested the absence of Weber's law. Specifically, for the conditions with feedback, the Bayes factors were *BF*_10_ = 0*.*33% ± 0*.*02%, *BF*_10_ = 0*.*26% ± 0*.*02%, and *BF*_10_ = 0*.*24% ± 0*.*02% for the visual feedback only (“V Fb”), haptic feedback (“H Fb”), and both visual and haptic feedback (“VH Fb”) conditions, respectively. Conversely, in “No Fb,” the results leaned toward the alternative hypothesis, suggesting a deviation from zero Weber's fraction. However, this evidence was only anecdotal, with a Bayes factor of *BF*_10_ = 1*.*27% ± 0*.*02%. These factors were derived based on a Cauchy distribution with a scale of 0.707, which was reasonable given that the standard deviations of Weber's fractions ranged from 0.07 to 0.09. Sensitivity analyses performed using rscales of 0.5 and 1.0, as well as traditional *t*-tests, yielded qualitatively similar results, as shown in Table C1 in Appendix C.

### Discussion

The results from this experiment suggest that pantomime grasping (without sensory feedback) adheres to Weber's law, even when visual information about the target is present throughout the movement and the grasp is directed at the same location as the target. In contrast, when online visual feedback or haptic feedback was available, grasping no longer followed Weber's law. Notably, it is a novel finding that visual feedback of the grasping fingers eliminates Weber's law.

## Experiment 2: Effect of sensory feedback on pantomime grasps

### Motivation

We aimed to further investigate *pantomime grasping*, defined in this paper as grasping without any sensory feedback throughout the movement, to gain additional insights into Weber's law in motor responses.

Although pantomime grasping is usually distinguished from “real” grasping, we hypothesize that they are not inherently different. Pantomime grasping can be made more similar to real grasping if its motor mapping becomes more aligned with that adapted with sensory feedback. To explore this, we intermixed pantomime grasps (equivalent to the “No Fb” condition in Experiment 1) with feedback grasps (equivalent to the “VH Fb” condition in Experiment 1) and examined the resulting change in the apparent Weber's fraction. This intermixing was intended to introduce the influence of sensory feedback without its direct presence, thus eliminating the possibility of online corrections. The influence can still take place such as through corrected biases and a more calibrated predictive model, meaning better alignment between motor commands and the resulting movement. Previous studies supported such influences, as grasps with sensory feedback showed significantly reduced systematic biases and improved precision, and pantomime grasps that were intermixed with these grasps also showed partial improvement in the same direction (Bingham et al., 2007; Bozzacchi, Volcic, & Domini, 2016). Furthermore, for grasps that were blocked rather than intermixed, the influence of sensory feedback continued into the following trials after its withdrawal if movements were not perturbed (Bozzacchi et al., 2014).

Our hypothesis predicted that, if these intermixed pantomime grasps show similar adjustments in motor outputs and adopt motor mappings more aligned with those of feedback grasps, then the apparent Weber's fraction will be reduced. As a control, we also included a condition where pantomime grasps and feedback grasps were performed in separate blocks.

### Methods

#### Design

In this experiment, participants were separated into two groups. Both groups of participants performed two types of grasps corresponding to the least and the most feedback: pantomime grasps with neither visual nor haptic feedback (“No fb”) and feedback grasps with both visual and haptic feedback (“VH Fb”). For one group of participants, pantomime grasps and feedback grasps were tested in different blocks. For the other group, feedback grasps and pantomime grasps were interleaved.

To sum up, the experiment had a 2 × 2 mixed factorial design (as shown in Figure 7): The first factor was the between-subject blocking condition—whether different grasps were organized in a BLOCKED or INTERMIXED manner. The second factor was the within-subject feedback condition—pantomime grasps versus feedback grasps. Importantly, both groups of participants were prompted at the beginning of each trial about whether sensory feedback would be available.

Subjects from the BLOCKED group were informed at the beginning of each block. Subjects from the INTERMIXED group were informed through an audio signal at the beginning of each trial.

#### Procedure for the BLOCKED group

Two types of grasps were completed in separate blocks. About half of the participants performed grasps with feedback first, and the other half performed grasps with no feedback first. The presentation order of object size was pseudorandomized. Before the initial grasping trial in each block, an auditory signal informed the feedback condition of the subsequent grasps.

In addition to reaching trials, to further reduce the carryover effect, two grasping blocks were separated by a block of distractor trials of manual size estimation. The estimations were performed either at the target (onsite) or to the right of home (offsite). With 10 repetitions at each location with each object size, the distractor block consisted of 80 trials. The order of estimation location and object size was pseudorandomized. Participants took a 1- to 5-minute break after each grasping block or distractor block.

#### Procedure for the INTERMIXED group

Grasps of different feedback conditions were intermixed. The trial order of the feedback condition and object size were pseudorandomized. At the beginning of each trial, an auditory signal informed the participant of the feedback condition, presented simultaneously with the visual stimulus.

Optional breaks were available every 40 trials of grasps.

#### Participants

Forty-three participants completed the BLOCKED condition, in which 20 performed the pantomime grasps first and 23 performed the feedback grasps first. Twenty participants completed the INTERMIXED condition. The participant number of the BLOCKED group was twice as many as the INTERMIXED group because we assumed an order effect for the BLOCKED group, which was found not to be significant in later statistical analyses.

Five participants from the BLOCKED group and two participants from the INTERMIXED group were identified as outliers and eliminated. For the data from the remaining participants, less than 3% of the responses were identified as outliers and removed.

### Results

**Figure 8.**
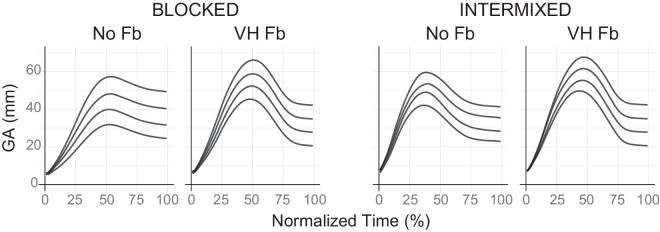
Average trajectories along normalized movement time in Experiment 2. For each condition, the trajectories ordered from lowest to highest correspond to object sizes of 21 mm, 28 mm, 35 mm, and 42 mm.

Figure 8 illustrates that trajectories varied between feedback conditions, with pantomime grasps (“No Fb”) being straighter. This difference was more pronounced when feedback conditions were blocked (left panel). When feedback conditions were intermixed (right panel), pantomime grasp trajectories became more similar to those of feedback grasps.

**Figure 9.**
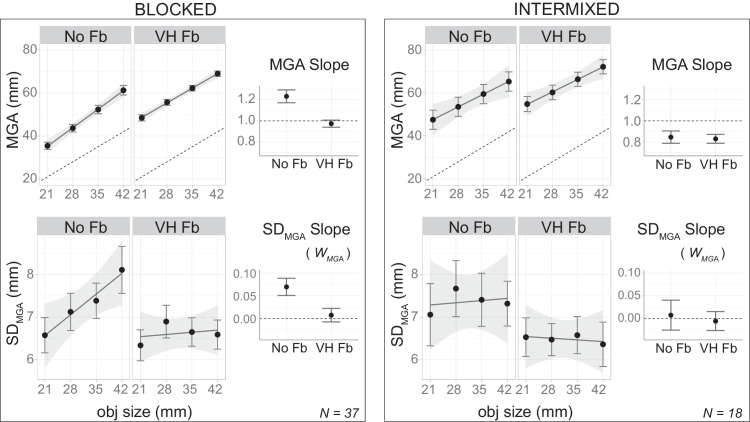
Aggregated results for Experiment 2. The left and right halves show results from separate groups of subjects: In the BLOCKED group, pantomime grasps (“No Fb”) and feedback grasps (“VH Fb”) were performed in separated blocks, whereas, in the INTERMIXED group, these grasps were intermixed. Within each half, the top row shows results regarding MGA and the bottom row shows results of *SD_MGA_*. Error bars indicate the standard errors of the mean across subjects.

Figure 9 illustrates that the response MGA also depended on the feedback condition. As shown in the upper row of the BLOCKED panel (left), blocked feedback grasps corresponded to a larger magnitude and a lower slope of the MGA compared with blocked pantomime grasps. When feedback conditions were intermixed (right panel), those differences were reduced. For these intermixed pantomime grasps, the response slope was not significantly different from that of intermixed feedback grasps and was lower than that of blocked pantomime grasps (see Table C2 in Appendix C).

#### Effect of feedback on Weber's fractions

Weber's fractions are illustrated in the bottom right corners of the BLOCKED (left) and INTERMIXED (right) panels in Figure 9. To estimate its change with sensory feedback, we calculated the within-subject difference between the condition “VH Fb” and the condition “No Fb” for each group. In the BLOCKED group (left panel), sensory feedback reduced Weber's fraction by an average of 0.062, a statistically significant reduction, *t*(36) = −2*.*687, *p* = 0*.*011, with a moderate effect size (Cohen's *d* = −0*.*442). In contrast, the INTERMIXED group (right panel) showed an average difference of –0.013, which was not significant, *t*(17) = −0*.*378, *p* = 0*.*710, and had a negligible effect size (Cohen's *d* = −0*.*089).

Additionally, to estimate the effect of intermixing on pantomime grasps, we compared Weber's fractions between the groups (“No Fb” from left vs. right panels of Figure 9). The Weber's fraction of the INTERMIXED group was 0.062 lower than that of the BLOCKED group. Although this difference was only marginally significant, *t*(53) = 1*.*751, *p* = 0*.*086, the effect size was moderate (*d* = 0*.*503), comparable to the change observed with sensory feedback.

#### Weber's law adherence in each condition

Data from the BLOCKED group (bottom right corner of the left panel of Figure 9) suggested that, when completed in separate blocks, pantomime grasps (“No Fb”) adhered to Weber's law (*BF*_10_ = 39*.*17% ± 0%), whereas feedback grasps (“VH Fb”) did not (*BF*_10_ = 0*.*20% ± 0*.*04%). Conversely, data from the INTERMIXED group (bottom right corner of the right panel of Figure 9) suggested that pantomime grasps did not adhere to Weber's law (*BF*_10_ = 0*.*24% ± 0*.*01%) when intermixed with feedback grasps, nor did those with feedback-grasps (*BF*_10_ = 0*.*25% ± 0*.*01%).

The Bayes factors were based on a Cauchy prior with an rscale of 0.717. Additional test results from traditional *t*-tests and from Bayes factor *t*-tests with different rscales, listed in Appendix Table C1, support the same hypothesis.

### Discussion

Results from the BLOCKED group replicated the typical finding that Weber's law is present in pantomime grasps but is absent in grasps with feedback. However, results from the INTERMIXED group suggested that this typical presence of Weber's law in pantomime grasps can be eliminated when they are intermixed with feedback grasps. Notably, participants were *aware* of the absence of sensory feedback before pantomime grasps were executed.

The findings from the INTERMIXED condition, coupled with the similarity in grip trajectories between pantomime and feedback grasps (Figure 8), suggest that the effect of sensory feedback was mediated through modified motor mapping: As sensory feedback induces error signals and updates the parameters of the motor program (e.g., the mapping between motor commands and motor outcomes), the motor mapping of pantomime grasps was modified.

The explanation based on the TVSH cannot account for our observations, as the TVSH proposes a relatively encapsulated sensorimotor system where error minimization and movement-updating processes are reserved for the dorsal stream (Whitwell, Buckingham, Enns, Chouinard, & Goodale, 2016). As the hypothesis also suggests that pantomime grasping is perceptual and processed by the ventral stream, these motor correction mechanisms should minimally affect the response.

Finally, it is important to note that visual feedback and haptic feedback, which were simultaneously present or absent in this experiment, operate differently. Visual feedback affects both the current movement and future movements, whereas haptic feedback only influences future movements. Further studies are needed to explore their distinct effects on Weber's law in action.

## Experiment 3: Effect of visual feedback on MSE

### Motivation

In the last experiment, we further examined the influence of sensory feedback on the adherence to Weber's law on manual size estimate. Previous studies have reported that this typical perceptual task exhibited Weber's law even when sensory feedback was provided. We aimed to explore in more detail whether MSE is indeed refractory to the influence of sensory feedback. Although past experiments reported Weber's law presence only after feedback compensation, we assessed changes in Weber's fraction with feedback.

Furthermore, we modified the delivery of sensory feedback to enhance its effectiveness. In previous studies, manual estimation was typically made near the body, away from the target object location. After completing the estimation, subjects needed to reach to grasp the target object to obtain haptic feedback, potentially reducing its effectiveness. Temporally, feedback was provided long after MSE completion, which is suboptimal for error correction mechanisms, based on sensorimotor adaptation research (Held, Efstathiou, & Greene, 1966; Martin, Keating, Goodkin, Bastian, & Thach, 1996; Welch, 1971). Spatially, feedback was delivered at a different location than where the MSE response was made, which might reduce the effect of feedback (Jazi & Heath, 2017), as discussed earlier.

**Figure 10.**
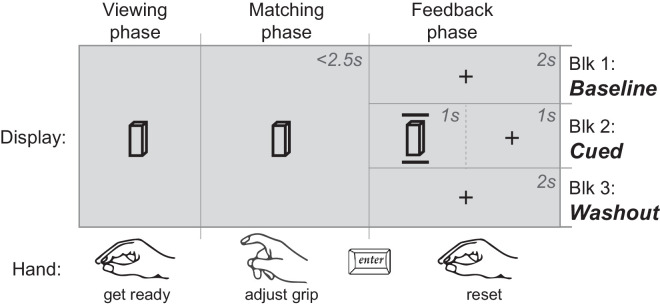
Trial procedure. The participant started with fingers pinched together at the target position. As the visual object became visible, they opened their fingers to match the height of the stimulus. The response was confirmed by a key press. A visual cue representing the entered MSE was presented in the following 1 second when the feedback was available (block 2); otherwise, a fixation cross was presented (blocks 1 and 3).

Admittedly, incorporating timely sensory feedback in manual estimation proves challenging. Presenting immediate haptic feedback after the response is difficult; maintaining online visual feedback would render the estimation task trivial. Eventually, we chose to present a simple visual cue: two horizontal bars positioned around the target object (see Figure 10). The bars appeared upon the completion of estimation for a brief period, with the distance between them representing the magnitude of the MSE, visualizing the error in the response. Furthermore, to avoid spatial dissociation between the target object and the feedback, the estimation was performed at the target position.

Although the visual cue in this experiment contained far less information compared with the ones in the previous grasping experiment—representing the grip aperture at a single time frame and not reflecting the orientation of the fingers—it still provided performance error, potentially leading to adaptation in motor mapping and changes in the apparent Weber's fraction.

### Methods

#### Design

The experiment consisted of three blocks of estimation trials (see Figure 10). In the first block, participants performed the estimation without receiving any feedback. In the second block, a visual cue appeared at every trial that indicated the discrepancy between the completed MSE and the actual object size. The third block was identical to the first block.

Within each block were 80 trials, with 20 repetitions for each object size. The presentation order of the object sizes was pseudorandomized. As subjects might tend to spend more time adjusting the grip aperture in the block where the visual cue is present, we imposed a 2.5-second time limit to ensure similar response time across conditions.

#### Procedure

MSE was performed at the target position, where the visual stimulus was displayed. The participant did not need to return home at the end of each trial but was asked to start a trial with fingers closed together. The subject was expected to complete the task within 2.5 seconds, starting from the presentation of the visual stimulus. If the key was pressed outside this limit, the participant received an auditory signal saying “faster.” In this case, they needed to close their fingers and repeat the MSE until the time constraint was met.

In the second block of trials, a visual cue showing the performed MSE appeared in the proximity of the target object for 1 second. The visual cue consisted of two horizontal lines 16 mm long located at equal distances from the top and bottom edges of the object. In this way, the participants could readily assess the error affecting their MSE. The visual cue was followed by a fixation cross for the duration of 1 second. In other blocks, the subject did not receive the visual cue. Instead, they viewed a fixation cross for 2 seconds (see Figure 10).

#### Participants

Thirty-three participants completed the experiment. Data from three outliers participants were eliminated. For the data from the remaining participants, less than 1% of the responses were identified as outliers and removed.

### Results

In a prescreening of the data, we eliminated from the analysis the first two repetitions of each block, as we observed drastic changes at the beginning of the second and the third block, when participants experienced sudden introduction and withdrawal of the visual cue (see Figure A2).

**Figure 11.**
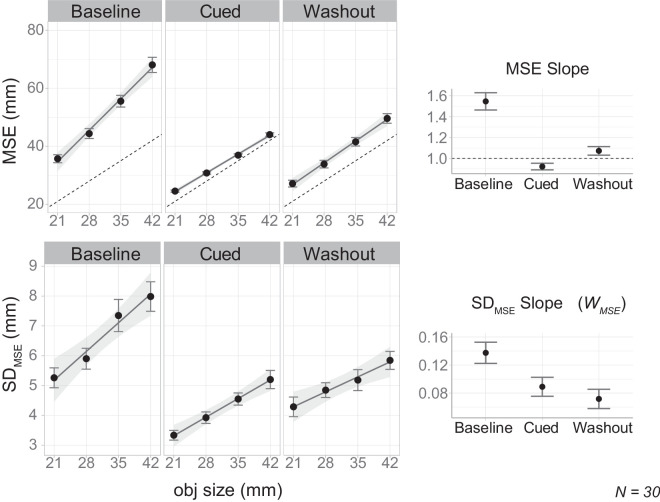
Aggregated results for Experiment 3. The top row shows results regarding MSE and the bottom row shows results for *SD_MSE_*. For each row, the left panels present the key variable as a function of object size in the baseline, cued, and washout blocks; the rightmost panel presents the slope of that variable with respect to object size. Error bars indicate the standard errors of the mean across subjects.

Figure 11 shows the average MSE (top left) and *SD_MSE_* (bottom left) as function of object size in the three experimental blocks. The right panels of Figure 11 show the slope of these variables in the three experimental blocks. To assess the influence of the visual cue, we estimated the change of Weber's fraction from the baseline block to the cued block. This difference was –0.048 ± 0.018, which is statistically significant, *t*(29) = −2*.*764*, p* = 0*.*001, with Cohen's *d* = −0*.*50. The estimated change from the cued to washout block was –0.017 ± 0.018, which is not statistically significant, *t*(29) = −0*.*937*, p* = 0*.*356, with Cohen's *d* = −0*.*17. In all conditions, Weber's law was present (Bayes factor *>* 1000) (see detailed test statistics listed in Table C1).

### Discussion

The results of this experiment show that effective visual feedback leads to a substantial reduction in both the variability magnitude and the apparent Weber's fraction of MSE responses. Moreover, we also showed that this reduction was sustained in a washout block where the feedback had been removed.

Unlike the previous two experiments, the introduction of visual feedback could not completely eliminate Weber's law. The persistent adherence to Weber's law for MSE is consistent with earlier findings and may indicate a fundamental distinction between manual estimation and grasping.

Nevertheless, there were some noteworthy differences between the MSE and the grasping tasks in the current study. First, the baseline Weber's fraction in this experiment was much higher compared with the grasping experiments. Second, the visual cue in this study provided significantly less information than the online visual feedback used in previous grasping experiments. Both factors may have contributed to the lingering presence of Weber's law in MSE, a point we explore further later.

Despite the only partial reduction of Weber's law, our finding suggests that when the appropriate feedback is provided, even a perceptual match is subject to motor adaptation when involving motoric effectors.

## General discussion

### Results summary

The results of the three experiments show that Weber's law associated with motor outputs, whether from an action task (reaching to grasp target objects) or a perceptual task (matching felt grip aperture to perceived object size), is modulated by sensory feedback. The results can be summarized as follows:

• When a grasp is executed without sensory feedback (pantomime grasping), the maximum grip aperture follows Weber's law.
• The introduction of either online visual feedback during execution or haptic feedback at the end of a grasping movement eliminates Weber's law.
• The elimination of Weber's law can be achieved even when sensory feedback is absent in the test grasps, as long as sensory feedback is available in other grasps that are intermixed with the test grasps.
• For manual estimation, a timely visual cue indicating estimation error significantly reduces the apparent Weber's fraction, although it does not eliminate Weber's law.

In the following sections, we further explore the potential mechanisms responsible for the observed reduction or elimination of Weber's law in motor tasks. We propose that the introduction of sensory feedback induces trial-by-trial motor learning and adaptation, remapping the motor output. With further analysis, we found converging evidence for this change in both grasping actions and manual size estimation. We then describe a simple mathematical model that could potentially explain in a qualitative fashion the empirical findings.

### Modified motor mapping by sensory feedback

#### Consistent slope decrease

A notable effect of sensory feedback across experiments was the decreased slope with respect to object size, a change that was statistically significant as detailed in Table C2 in Appendix C.

This change is likely due to modifications in motor mapping rather than visual estimation. Sensory feedback provides error signals—whether sensory error or performance error—that alter the mapping between visual estimates and motor outputs. The slope decrease in MSE was not surprising as the baseline was much higher than the veridical value, and the feedback helped correct this inflation. Conversely, the consistent decrease in the slope of MGA of grasps—whether induced by online visual feedback or final haptic feedback, or both—is intriguing. Critically, with sensory feedback, the slope tended to be lowered to values smaller than the veridical value of 1, indicating the change was prompted to promote movement efficiency rather than to improve size estimation. A smaller slope means less variable spatiotemporal trajectories as object size varies, which is preferable for energy efficiency. The consistent decrease in slope indicated a general mechanism of error correction, through which movement trajectories converge to more efficient grasping patterns.

#### Trial-by-trial change

**Figure A1.**
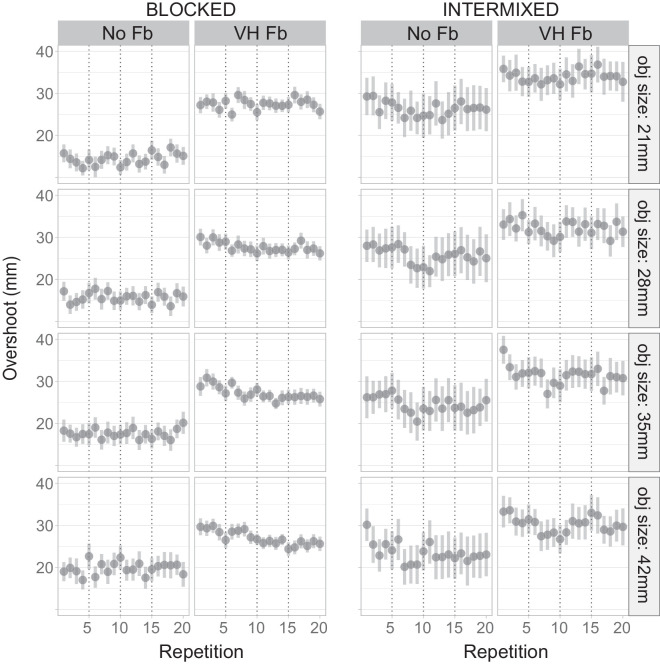
Temporal evolution of MGA overshoot in Experiment 2. The MGA overshoot, defined as the amount by which the MGA exceeds the object size, is plotted as a function of repetition for each object size and feedback condition. The left two columns represent the BLOCKED group results, and the right two columns represent the INTERMIXED group results. Vertical dotted lines in the plots indicate breaks (afterward), which might have introduced discontinuity. All error bars indicate between-subject standard errors of the mean.

Temporal analyses of response (shown in Appendix A) also provided additional insight into the effect of sensory feedback on motor responses. Specifically, the temporal change of the MGA in Experiment 2 (characterized by MGA overshoot, or its difference from the object size) provided anecdotal evidence of the trial-by-trial correction caused by sensory feedback (Figure A1). Whereas adaptation over repeated movement was expected in feedback grasps (grasps where visual and haptic feedback were both available), pantomime grasps intermixed with feedback grasps also exhibited this adaptation pattern.

**Figure A2.**
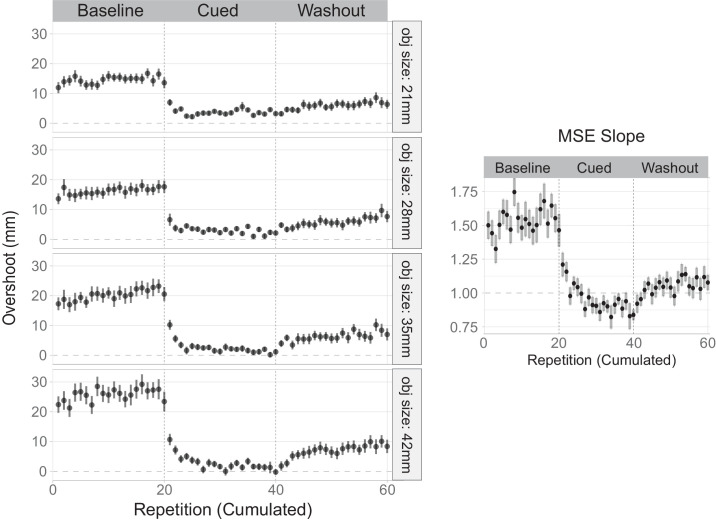
Temporal change of MSE in Experiment 3. The left panel shows the temporal change in MSE overshoot, defined as the amount by which the MSE exceeds the object size. It is plotted as a function of repetition for each object size and feedback condition. The right panel illustrates the temporal change in the MSE slope with respect to object size. Vertical dotted lines indicate breaks (after), which might have introduced discontinuity. Horizontal dashed lines represent veridical values. Error bars indicate between-subject standard errors of the mean.

The trial-by-trial correction was more evident in the temporal evolution of the MSE in Experiment 3 (Figure A2), as MSE was less noisy compared with the response from grasps. In the baseline block, where no feedback was provided, there was no significant change across repetitions, for either MSE (Figure A2, left panels) or MSE slope (Figure A2, right panel). Notably, the baseline slope was so high that it was unlikely due to the inflated scale of the visual estimate, given that the estimate measured using other tasks typically reports a slope close to 1. Instead, the inflation likely stemmed from distorted motor mapping with uncalibrated proprioception. However, in the cued block, as the cue was introduced, the MSE and its slope rapidly converged toward the veridical value over the first numerous repetitions and then stabilized around the veridical value for the rest of the trials. Critically, in the washout block where feedback was withdrawn, despite some drift back to the baseline values most of the effect of the cue was sustained, as the washout MSE and slope remained closer to the values at the end of the cued block than to the baseline values.

It is worth highlighting that the temporal changes in the cued and washout blocks resembled those typically observed in sensorimotor adaptation, indicative of similar mechanisms (e.g., trial-by-trial movement updating and error correction). These observations appear at odds with the TVSH claim that posits a relatively encapsulated sensorimotor system to which the ventral stream has limited access. However, if dorsal stream processing were activated, the hypothesis would expect an absence of Weber's law in MSE, which did not align with our observations.

#### Interpretation

Collectively, our observations suggest that a significant portion of the influence of sensory feedback on our responses was mediated through modified motor mapping accumulated over trials. One key support comes from the MSE observations, where the modifications in response mapping from the cued block persisted into the washout block. This helps explain the results of pantomime grasps that were intermixed with feedback trials: When sensory feedback modified the motor mapping based on error signals, the modification affected subsequent movements even after feedback was withdrawn. Therefore, in the INTERMIXED condition, as feedback grasps underwent trial-by-trial motor adaptation, the adapted motor mapping applied to following pantomime grasps.

Additionally, the persistence of the feedback effect may explain the inconsistencies in the (blocked) pantomime grasps from Experiment 1 and Experiment 2. Despite employing breaks and distractor trials intended to counteract carryover effects, subsequent grasping appeared influenced. Experiment 1 contained more grasping blocks with feedback than Experiment 2, making the pantomime grasping in Experiment 1 more likely to be affected by carryover effects from previous feedback, which might explain its lower MGA slope and lower Weber's fraction compared with the counterparts in Experiment 2.

In summary, our observations suggest that sensory feedback drove trial-by-trial motor mapping adjustments via error signals. This modification led to reduced response slopes across conditions where feedback was available. Under the influence of feedback, MSE was improved, but the MGA of grasps became less responsive to size changes. The next section discusses how this impacts Weber's fraction.

### Potential mechanisms for the reduced Weber's fraction with sensory feedback

**Figure 12.**
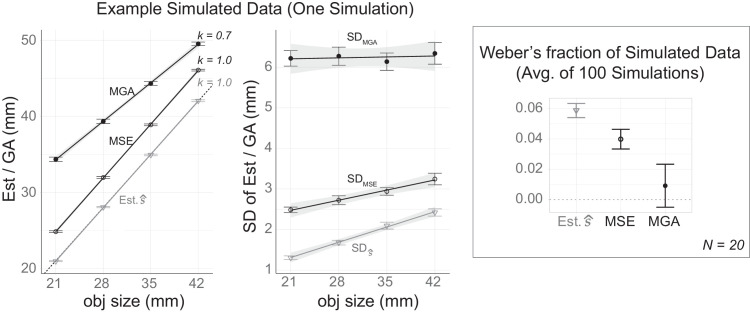
Simulated experiments comparing MSE and MGA based on the same visual estimates. Both MSE and MGA were derived from the same visual estimates, simulated with a Weber's fraction of 0.06: $\hat{s}=s+\varepsilon_{V}$, where $\varepsilon_{V}∼N\left(0,{\left(0.06s\right)}^{2}\right)$. However, the two metrics differ in their mapping slopes, implementation noise levels, and overshoot values (with overshoot differences being less central to the discussion). MGA is characterized by a compressed mapping slope of 0.7, a large overshoot of 18 mm, and greater implementation noise (σ=6): $MGA=0.7\cdot \hat{s}+18+\varepsilon_{i}$, where $\varepsilon_{i}∼N\left(0,6^{2}\right)$; In contrast, MSE assumes a unity mapping slope of 1.0, a smaller overshoot of 4 mm, and lower implementation noise (σ=2): $MSE=\hat{s}+4+\varepsilon_{j}$, where $\varepsilon_{j}∼N\left(0,2^{2}\right)$.

We compared these two tasks by simulating experiments with 20 hypothetical subjects, each completing 20 repetitions per condition (as shown in Figure 12). On the left we present the data from one simulated experiment as an example. On the right, we present the average Weber's fraction from 100 simulations. All error bars indicate between-subject standard errors of the mean. The simulated results suggest that MGA, which corresponds to a smaller mapping slope and greater noise, tends to yield a smaller Weber's fraction compared with the MSE even when they are both based on the same visual estimate.

Consider again Figure 1, where we represent any task involving a motor execution as a two-stage process. In the first stage, the visual system produces a visual estimate *ŝ* of the quantity of interest *s*. The second stage establishes a mapping *f*(*ŝ*) between this estimate and the motor response. In the simplest case, we assume that this function is linear:

$$\begin{array}{c} GA^{*}=f\left(\hat{s}\right)=k\cdot \underset{\mathrm{Estimate}}{\underset{︸}{\hat{s}}}+\underset{\mathrm{Motor}\;\mathrm{mapping}}{\underset{︸}{overshoot}} \end{array}$$

where *k* is the slope of the response function and *overshoot* is the intercept. For grasping actions, *overshoot* refers to the safety margin typical of the MGA. We can now assume two sources of noise that may affect the GA*. The first is the noise affecting the visual estimate *ŝ* with variance σ^2^. Assuming that the noise of the visual estimate obeys Weber's law: σ_*ŝ*_ = *W_V_* × *s*, where *W_V_* is the Weber fraction. The second source of noise is the motor noise (standard deviation σ*_overshoot_*) associated with *implementation* of the motor command (e.g., the variability of the safety margin of the MGA). If we further assume that these two sources of noise are independent, we can calculate the standard deviation of the grip aperture:

$$SD_{GA^{*}}=\sqrt{k^{2}\cdot {\underset{︸}{{\left(W_{v}\cdot s\right)}^{2}}}_{\mathrm{Estimate}}\;+\;{\underset{︸}{\sigma^{2}overshoot}}_{\mathrm{Motor}\;\mathrm{mapping}}}$$

This model of the noise shows that SD_GA__*_ is not a good read-out of the estimation noise *W_V_* × *s* because (a) the estimation noise is modulated by the slope *k* of the response function; and (2) other forms of variability (e.g., temporal changes, motor noise in execution) contribute to the observed variability, further obscuring the true pattern of estimation noise. Using *SD_MGA_* of grasps to probe the pattern of visual estimates is particularly futile, due to the combination of low response slopes and high implementation noise. An analogy for this attempt would be listening to a car radio while driving on a highway, with the volume turned down and the car windows open.

But it is important to note, however, that the analogy is not accurate when it comes to the combination of different sources of noise. Unlike a direct addition in the radio example, the combination of noise terms that form SD_GA__*_ is not linear, meaning the contribution from each part is not proportional. Between the estimation noise and other noise outside of the estimate, whichever takes dominance will overshadow the influence of the other term.

First and foremost, a decrease in the slope *k* of the response function tunes down the contribution from the estimation noise to the observed scalar variability of the *GA*. Second, because the magnitude of the additional noise is most likely larger than the magnitude of the estimation noise, a low value of *k* would render the influence of the estimation noise negligible. Consequently, Weber's law would not be detectable in the motor output. Indeed, our simulations assuming that *W_V_* is 0.06^3^ (see Figure 12) suggest that MGA can fail to show Weber's law even when the underlying visual estimates follow the law. Instead, the Weber law is observed in simulated MSE responses because, in this case, the slope of the motor mapping is larger and the noise affecting the MSE is smaller.

Another possible mechanism of the effect of sensory feedback on the scalar variability of the GA* is the changed linearity of the mapping function. According to a recent research (Bhatia et al., 2022), the apparent Weber's fraction in grasping could be related to nonlinearities in the scaling of the grasping response. Specifically, compared with linear mapping, responses mapped through a convex function with respect to object size are more likely to adhere to Weber's law, whereas responses mapped through a concave function are less likely to do so.

Preliminary analyses of nonlinearity in our data suggest that the mapping function tends to be more convex when no feedback is available, but it tends to become more concave when feedback is introduced (see Appendices for more details). This shift toward concavity with feedback could explain the reduced adherence to Weber's law observed in conditions with sensory feedback. This aligns with the notion that sensory feedback induces changes in motor mapping, which in turn affects the variability of the motor response.

Whether the modulation of the scalar variability of the GA* caused by sensory feedback is due to a change in the overall slope of the grasping response or a change in the response convexity, or both, is a matter of future research. Critically, the observed changes in motor mapping alone can account for the reduction of Weber's fraction, offering a simpler explanation than the prevalent explanation that is based on dissociated visual processing.

### General implications

The experiments in the current paper investigated the hypothesis that the modification of motor mapping induced by sensory feedback in grasping can account for the uncharacteristic response variability, providing a motor-based explanation for the absence of Weber's law in grasping. This serves as an alternative to the prevalent vision-based explanation. The findings of three experiments provide converging support for this hypothesis.

More broadly, the hypothesized mechanism—feedback-driven motor adaptation—has also been used to explain other instances where action responses differ significantly from perceptual responses (Cesanek, Campagnoli, Taylor, & Domini, 2018; Kopiske, Cesanek, Campagnoli, & Domini, 2017; Schenk, 2012). Collectively, the systematic differences in the availability of sensory feedback play a significant role in differentiating perception and action observed in behavioral data.

Our experiments were built to test a novel hypothesis that is distinct from existing explanations. However, some of our findings may also be noteworthy for other explanations:

• From the perspective of the TVSH explanation, our findings suggest that real-time visual information of the target object is not sufficient to activate dorsal stream processing. Instead, effective error signals may be the key, as indicated by our results and those reported by Jazi and Heath (2017).
• The MGA of the pantomime grasps from Experiment 2 and MSE from Experiment 3 appeared to be influenced by error minimization and movement-updating processes, which seem at odds with the TVSH claim that these processes are reserved for dorsal processing.
• In Experiment 1, the MGA in grasps with visual feedback only (“V Fb”) was smaller than in grasps with no feedback (“No Fb”), yet Weber's law was absent in the former but present in the latter. This particular pair of observations may not be accounted for by the biomechanical limit alone.

As a final consideration, our findings suggest that controlling the influence of sensory feedback across tasks could be challenging. To effectively facilitate the impact of sensory feedback on responses, both the temporal and spatial aspects of feedback delivery must be considered. The mere presence of sensory feedback may not be sufficient. Effective feedback may need to be timely and spatially aligned with the task to trigger the desired error correction and motor adaptation mechanisms. Future research could delve deeper into the spatial and temporal factors that modulate the influence of sensory feedback on motor actions, thereby unveiling the intricate mechanisms underlying this intriguing phenomenon.

## Conclusions

We examined grasping and manual estimation under various feedback conditions and found that both responses are influenced by sensory feedback, altering the mapping between physical input and motor output. This mapping change reduced the apparent Weber's fraction, suggesting an alternative explanation for the absence of Weber's law in grasping that does not require different visual processing. Furthermore, we question whether Weber's fraction derived from MGA reflects visual estimation noise. Schenk et al. (2017) argued that making a secure and firm grip involves multiple factors beyond size estimation, such as forces, energy, and comfort. These factors introduce variability in motor actions that likely overshadow the contribution of visual estimates.

## Figures and Tables

**Figure B1.**
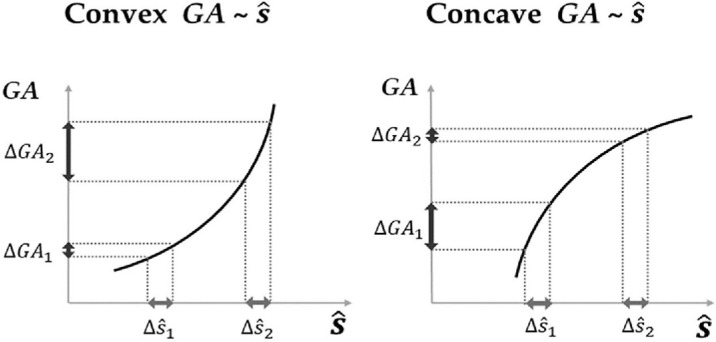
This figure was adapted from Bhatia et al. (2022) to illustrate how convexity/concavity influences *SD_MGA_*. The horizontal axes denote the size estimate, and the vertical axes denote the mapped GA*. Given the same amount of estimation noise at small and at large objects, a convex map tends to inflate Weber's law, and a concave map tends to reverse Weber's law.

**Figure B2.**
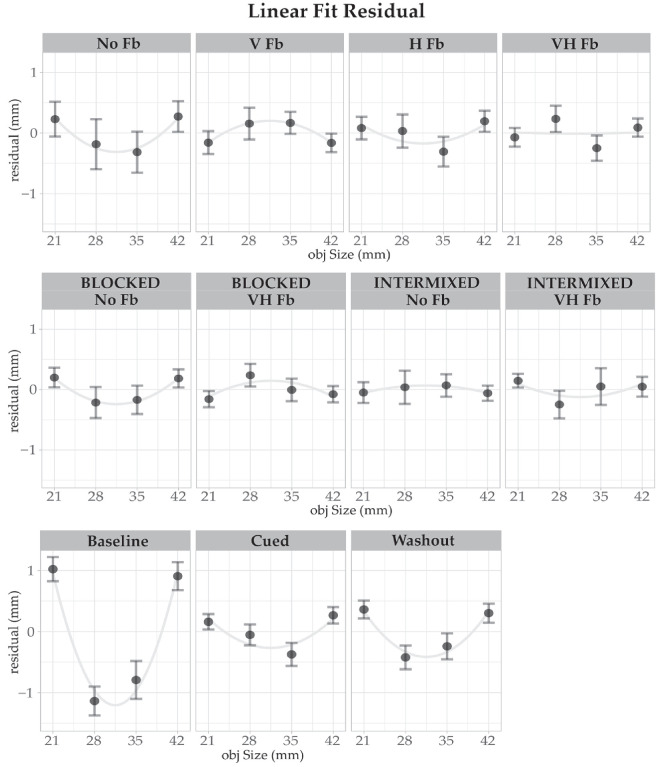
Residuals of the linear fits on the GA* as a function of object size in the three experiments (rows) in all experimental conditions (columns). Notably, the residual functions are convex when sensory feedback is absent. This convexity is reduced or reversed when sensory feedback is present.

## Footnotes

1 “Ventral processing” and “dorsal processing” have become more general terms that are less tied to specific neural pathways. Ventral-processing estimates, for example, can refer to estimates that are characteristic of perception. Nevertheless, the use of the terms still implies (a) visual estimates for perception and action are processed differently, making perceptual and action estimates distinct, and (b) it is the distinctions in the visual estimates that lead to characteristic differences in their responses. In the current paper, we use *processing stream* and *processing mode* interchangeably.

2 Pantomime grasping was originally defined by Liepmann in 1905 as grasping without physical interaction with the target object. Now it is also known as simulated grasping or pretended grasping and is a general term used in various contexts, including (a) grasping directed at an area adjacent to the physical object (for example, in the study by Holmes, Lohmus, McKinnon, Mulla, & Heath, 2013, subjects were instructed to complete their response as if the object were located at that designated area); (b) grasping with the physical object removed before movement onset (e.g., Davarpanah Jazi, Yau, Westwood, & Heath, 2015); and (c) grasping toward a virtual object, such as the mirror reflection of a physical object (e.g., Bingham, Coats, & Mon-Williams, 2007). In the current study, we refer to pantomime grasping as grasping with no sensory feedback throughout the movement—specifically, neither online visual feedback nor final tactical feedback. It is important to note, however, that our visual stimulus remained visible throughout the movement, providing continuous visual information about the target object during both motor programming and execution.

3 This Weber's fraction is based on previously reported values (Mckee & Welch, 1992; Ono, 1967; Van Beers, Sittig, & Denier van der Gon, 1998). It is also the value used by other researchers for simulation (Smeets & Brenner, 2008).

## Appendix A: Temporal effect

### Experiment 2

We observed^13^,^14^ a potential contrast: For pantomime grasps (“No Fb”), overshoots did not exhibit notable changes in the BLOCKED group, whereas they appeared to progressively decrease in earlier repetitions in the INTERMIXED group, indicating adaptation over repetitions. This decreasing pattern was also observed in feedback grasps (“VH Fb”), whether BLOCKED or INTERMIXED. However, because the data exhibited considerable variability, more data are required to confirm these observations.

### Experiment 3

In the baseline block, responses did not show notable changes over repetitions. Overshoots remained positive, and the MSE slope stayed inflated. As the visual cue became available in the cued block, preexisting biases were gradually corrected, with both overshoots and the MSE slope progressively decreasing until reaching equilibrium near the veridical value. After withdrawing the cue in the washout block, responses gradually drifted back to their baseline values; however, the correction of preexisting biases was mostly sustained.

## Appendix B: Nonlinear scaling of response function and Weber's law

This explanation comes from a recently published investigation relating the apparent disappearance of Weber's law in grasping to nonlinearities in the scaling of the grasping response (Bhatia et al., 2022). Consider Figure B1, where we show two possible kinds of nonlinear functions relating visual estimate to grip aperture. If we assume constant estimation noise, indicated by ∆*s_i_* (*i* = 1, 2) on the figure, then the nonlinearity in the scaling of the grasping response will yield variability of the *GA** depending on object size. If the function is convex (Figure B1, left), then the *GA** will adhere to Weber's law. In turn, if the function is concave (Figure B1, right), then the *GA** variability will exhibit the inverse of Weber's law (i.e., *GA** variability decreasing with object size). Let's now assume that the visual estimate is actually subject to Weber's law. In this case, a convex function (Figure B1, left) would exaggerate Weber's law, whereas the concave function (Figure B1, right) would counteract it with the effect of diminishing it or eliminating it.

To check whether this explanation applied to our results, we inspected the residuals of the linear fit on all responding grip apertures (see Figure B2).

Although the number of testing objects limited our ability to draw more definitive conclusions, we did observe a notable trend of converging changes, as the presence or absence of sensory feedback affected the sign of the observed deviation from linearity. In all experiments, the no-feedback conditions exhibited a convex nonlinearity. When feedback was present, this convexity was substantially reduced or, in some cases, reversed to concavity. As explained above, convexity exaggerates the scalar variability of the *GA**. Conversely, the reduction of convexity found in the feedback conditions predicts a reduction or elimination of Weber's law.

## Appendix C: Additional test results

See Tables C1, C2, and C3.

**Table C1.** T-test on Weber's fractions (*H*_0_ : *WB* = 0).

	*d*	*t*	*df*	*p*	*BFr* = 0*.*7	*BFr* = 0*.*5	*BFr* = 1*.*0	Weber's Law
“No Fb”	0.47	2.029	18	0.058	1.27	1.45	1.04	Yes
“V Fb”	−0.20	−0.858	18	0.402	0.33	0.42	0.25	No
“H Fb”	0.10	0.421	18	0.679	0.26	0.34	0.19	No
“VH Fb”	−0.04	−0.155	18	0.878	0.24	0.32	0.18	No
BLOCKED “No Fb”	0.60	3.672	36	0.001	39.18	40.57	34.47	Yes
BLOCKED “VH Fb”	0.08	0.495	36	0.623	0.20	0.27	0.14	No
INTERMIXED “No Fb”	0.05	0.222	17	0.827	0.25	0.33	0.18	No
INTERMIXED “VH Fb”	−0.06	−0.272	17	0.789	0.25	0.33	0.19	No
Baseline	1.66	9.083	29	*<*0.001	2.4 × 10^7^			Yes
Cued	1.20	6.567	29	*<*0.001	5.3 × 10^4^			Yes
Washout	0.95	5.194	29	*<*0.001	1.5 × 10^3^			Yes

**Table C2.** Testing differences in the mapping slope *k* of *GA*^*^ with respect to object size.

	Estimated	*d*	*t*	*df*	*p*
“V Fb” − “No Fb”	−0.235	−0.93	−4.07	18	*<*0.001*
“H Fb” − “No Fb”	−0.303	−1.08	−4.72	18	*<*0.001*
“VH Fb” − “No Fb”	−0.350	−1.35	−5.90	18	*<*0.001*
BLOCKED “VH Fb” − BLOCKED “No Fb”	−0.258	−0.71	−4.31	36	*<*0.001*
INTERMIXED “VH Fb” − INTERMIXED “No Fb”	−0.016	−0.10	−0.40	17	0.691
INTERMIXED “No Fb” − BLOCKED “No Fb”	−0.384	−1.15	−4.00	53	*<*0.001*
Cued − Baseline	−0.624	−1.51	−8.26	29	*<*0.001*
Washout − Cued	0.150	0.68	3.71	29	*<*0.001*

The asterisk *p*-values signify statistical significance at the 0.05 level.

**Table C3.** Testing differences in the magnitude of response variability *GA^*^_SD_* that was averaged across object sizes.

	Estimated	*d*	*t*	*df*	*p*
“V Fb − “No Fb”	−2.100	−0.74	−3.23	18	0.005*
“H Fb” − “No Fb”	−1.947	−0.76	−3.38	18	0.003*
“VH Fb” − “No Fb”	−2.364	−0.93	−4.06	18	*<*0.001*
BLOCKED “VH Fb” − BLOCKED “No Fb”	−0.680	−0.26	−1.58	36	0.124
INTERMIXED “VH Fb” − INTERMIXED “No Fb”	−0.878	−0.64	−2.71	17	0.015*
INTERMIXED “No Fb” − BLOCKED “No Fb”	0.065	0.03	0.09	53	0.926
Cued − Baseline	−2.371	−1.40	−7.65	29	*<*0.001*
Washout − Cued	0.788	0.74	4.06	29	*<*0.001*

The asterisk *p*-values signify statistical significance at the 0.05 level.
